# Medicare Part D and Cost-Sharing for Antiretroviral Therapy and Preexposure Prophylaxis

**DOI:** 10.1001/jamanetworkopen.2020.2739

**Published:** 2020-04-14

**Authors:** Chien-Wen Tseng, R. Adams Dudley, Randi Chen, Rochelle P. Walensky

**Affiliations:** 1 Department of Family Medicine and Community Health, John A. Burns School of Medicine, University of Hawaii, Honolulu; 2 Pacific Health Research and Education Institute, Honolulu, Hawaii; 3 School of Medicine, Institute for Health Informatics, University of Minnesota, Minneapolis; 4 School of Public Health, University of Minnesota, Minneapolis; 5 Center for Care Delivery and Outcomes Research, Minneapolis Veterans Affairs Medical Center, Minneapolis, Minnesota; 6 Medical Practice Evaluation Center, Division of Infectious Diseases, Massachusetts General Hospital, Boston; 7 Harvard University Center for AIDS Research, Harvard Medical School, Boston, Massachusetts

## Abstract

**Importance:**

The 2019 federal Ending the HIV Epidemic initiative requires a vast expansion of access to antiretroviral therapy (ART) and preexposure prophylaxis (PrEP) for HIV treatment and prevention. However, high prices for ART and PrEP can reduce their affordability and use. Medicare covers 1 in 4 persons living with HIV, and the Medicare Part D drug benefit imposes complicated cost-sharing between patients and other stakeholders.

**Objective:**

To determine how the Medicare Part D design distributes the cost burden for ART and PrEP between patients, insurance plans, manufacturers, and Medicare.

**Design and Setting:**

Nationwide cross-sectional analyses of first quarter 2019 Medicare formulary and pricing files for 3326 Part D plans were performed. These files contain drug benefit data, including prices and cost-sharing requirements.

**Main Outcomes and Measures:**

For 18 ART and 2 PrEP regimens, the out-of-pocket costs for patients and the cost borne by plans, manufacturers, and Medicare were projected for 1 year of treatment or prevention under a 2019 standard Medicare Part D insurance plan. Analyses assumed that patients used the ART or PrEP regimen and no other medications.

**Results:**

In 2019, ART prices ranged from $24 010 to $46 770 annually (median price, $35 780), with patients projected to pay 9% to 14% of the cost ($3270-$4350), insurance plans 18% to 24% ($5340-$8450), manufacturers 6% to 11% ($2370-$2750), and Medicare 53% to 67% ($12 770-$31 270). The price of PrEP was $20 570 annually, with patients contributing 15% ($2990), insurance plans 22% ($4570), manufacturers 13% ($2750), and Medicare 50% ($10 260). For beneficiaries with low-income subsidies that cover all patient cost-sharing, Medicare would assume 67% to 76% of ART costs and 65% of PrEP costs.

**Conclusions and Relevance:**

Medicare Part D mandates universal ART and PrEP coverage, but high prices (>$35 000 annually for ART and>$20 000 annually for PrEP) and the design of Part D can jeopardize affordability for patients and place most of the cost burden on taxpayers. Under a standard Medicare Part D benefit, patients pay $3000 to $4000 out-of-pocket yearly, unless they qualify for low-income subsidies, and half to two-thirds of the cost of ART and PrEP is borne by Medicare rather than insurance plans or manufacturers. To end the HIV epidemic by 2030, it appears that policies must address both high drug prices and revamp Medicare Part D cost-sharing.

## Key Points

**Question:**

How is the cost burden of antiretroviral therapy and preexposure prophylaxis for HIV treatment and prevention distributed to patients and other payers under Medicare Part D?

**Findings:**

In this nationwide cross-sectional analysis of 2019 Part D plans, the median prices of antiretroviral therapy exceeded $35 000 annually, and the price of preexposure prophylaxis exceeded $20 000 annually. Patients’ projected annual out-of-pocket cost surpassed $3000, and Medicare, not insurance plans or manufacturers, paid half to two-thirds of costs.

**Meaning:**

It appears that ending the HIV epidemic requires addressing the high prices of antiretroviral therapy and preexposure prophylaxis and Medicare Part D cost-sharing that jeopardize affordability for patients and incur a burden to Medicare.

## Teaser

This nationwide cross-sectional analysis examines how the Medicare Part D design distributes the cost burden for antiretroviral therapy and preexposure prophylaxis (PrEP) between patients, insurance plans, manufacturers, and Medicare.

## Introduction

More than 1 million Americans live with HIV and an additional 1.2 million individuals have indications that make them eligible for medications to reduce risk for acquiring HIV infection.^1,2,3^ Despite effective antiretroviral (ARV) drugs for treatment and prevention, approximately 38 000 persons are newly infected with HIV annually, a number remaining essentially unchanged since 2012.^1,2,3^ The 2019 federal Ending the HIV Epidemic initiative seeks a 75% reduction in new HIV infections by 2025 and a 90% reduction by 2030.^4^ The program’s success requires a rapid and substantial increase in the use of ARVs. Suppressive ARV therapy (ART) for persons with HIV is associated with undetectable virus levels, thus preventing transmission to others, and preexposure prophylaxis (PrEP) for persons at risk for HIV may be associated with a more than 90% lower risk of infection.^5^

Antiretroviral therapy and PrEP cost tens of thousands of dollars annually.^6,7^ Consequently, the financial burden to patients is a concerning barrier to expanding ARV use.^6,7,8,9,10,11^ Furthermore, ARV prices continue to increase steadily,^7,12,13,14^ with a 34% price increase between 2012 and 2018.^7^ High ARV prices also affect taxpayers. Historically, many people with HIV relied on Medicaid (federally or state-subsidized health coverage for lower-income persons) owing to disabilities from HIV prior to the availability of effective ART.^15^ However, Medicare, which covers persons aged 65 years or older or younger persons with permanent disabilities, has played an increasingly important role in federal funding for HIV care. In 2006, the Patient Protection and Affordable Care Act began automatically enrolling Medicaid beneficiaries with Social Security Disability Insurance into dual coverage with Medicare after 2 years.^15^ By 2014, approximately 1 in 4 persons with HIV who received care were insured in part through Medicare.^15^ Most Medicare beneficiaries with HIV qualify for coverage on disability alone (79% in 2014). However, an increasing number of individuals with HIV enter Medicare by reaching 65 years or older (13% in 2014) because ART enables people with HIV to live longer and age in a healthy manner.^15^

In this study, we examined cost-sharing for ART and PrEP under Medicare Part D, the drug benefit that covers 70% of Medicare beneficiaries.^16^ Medicare beneficiaries with HIV are even more likely to have Part D coverage because dually eligible individuals (Medicare and Medicaid) automatically receive Part D benefits.^15,16^ By federal mandate, Part D must cover all ARVs as 1 of 6 specially protected drug classes.^16^ However, as designed, Part D also imposes cost-sharing among patients, insurers, manufacturers, and Medicare that varies throughout the year.^16^ Studies for other conditions, such as cancer and rheumatoid arthritis, have shown that Part D enrollees can face substantial out-of-pocket costs for expensive drugs even when these drugs are covered by Part D.^17,18^ Patients must pay an initial deductible, followed by a coverage phase (insurance plans pay most of the cost, but patient cost-sharing is required), then a coverage gap (costs are shared between the patient, the insurance plan, and the manufacturer), and finally a catastrophic coverage phase (Medicare pays 80% of the cost, with the remainder shared by the insurance plan and the patient).^16^ For individuals with qualifying low income and assets and for dually eligible enrollees, Medicare provides a full or partial low-income subsidy (LIS) that covers all or part of the out-of-pocket costs.^16^ In 2014, 77% of Medicare beneficiaries with HIV received an LIS.^15^

This complicated Part D cost-sharing structure obscures who carries the cost burden for high-priced ART and PrEP. However, the way in which Part D distributes the high cost of ART and PrEP to its enrollees and to taxpayers may affect how much patients and society pay to prevent or control HIV. We analyzed how cost is shared between patients, insurance plans, manufacturers, and Medicare for 1 year of ART or PrEP, and how this cost-sharing would differ for patients qualifying for full taxpayer subsidies.

## Methods

We analyzed the first quarter 2019 Medicare formulary and pricing files for 3326 Part D insurance plans nationwide.^19^ Files contain drug benefit data for each insurance plan (formulary coverage, 30-day list prices for drugs, and copayments and coinsurance requirements) and not patient claims. We averaged prices and the out-of-pocket cost requirements for 18 first-line ART regimens based on US Department of Health and Human Services recommendations and 2 US Food and Drug Administration–approved PrEP regimens.^20^ This study was approved by the University of Hawaii Institutional Review Board and the Massachusetts General Hospital Partners Human Research Committee. The databases contain insurance plans’ drug benefit design information (no patient data), and therefore this study did not require informed consent.

For each regimen, we projected the proportion of annual treatment cost paid by the patient, insurance plan, manufacturer, and Medicare under a standard 2019 Part D insurance plan consisting of the following 4 phases^16^: (1) the patient deductible phase, fixed at $415; (2) the covered phase, in which patients pay part of the drug price (set as mean cost-sharing requirements by Part D plans nationwide), with the remaining cost paid by the insurance plan; (3) the “donut hole” or coverage gap: once total drug costs reach $3820, brand-name drug costs are shared by the patient (25%), manufacturer (70%), and insurance plan (5%), while generic drug costs are shared by the patient (37%) and manufacturer (63%); and (4) catastrophic coverage: after out-of-pocket costs (including manufacturers’ discounts for brand-name drugs during the gap) total $5100, costs are borne by the patient (5%), insurance plan (15%), and Medicare (80%) until the end of the year. Part D bases all coinsurance cost-sharing on a drug’s full list price, which excludes rebates and discounts to plans.^21^ For each monthly prescription, we prorated the proportion of drug price falling into each Part D phase and calculated cost-sharing accordingly. We also considered a full LIS scenario, in which Medicare absorbs virtually all of a patient’s out-of-pocket costs for ART and PrEP throughout the year.^22^

Analyses were based on patients using the specific ART or PrEP regimen and no other drugs. We used simple descriptive statistics to present mean monthly prices and out-of-pocket cost requirements across all plans, rounded to the nearest $10.

## Results

In 2019, mean (SD) monthly prices of ART ranged from $2000 ($140) to $3900 ($110), with a median monthly price of $2980, and the mean (SD) monthly price of PrEP was $1710 ($60) (Table 1). During the coverage phase, insurance plans almost universally structured patient cost-sharing as a coinsurance (ie, a percentage of a drug’s list price [range, 28.1%-30.8%] rather than charging a fixed dollar copayment). This cost-sharing structure parallels the coinsurance required in the coverage gap (25%) and catastrophic (5%) phases. For ART, the monthly out-of-pocket costs after the deductible were high in the coverage phase (mean [SD]: range, $550 [$160]-$1160 [$210]) and coverage gap phase (range, $530-$970) and remained considerable even after reaching catastrophic coverage (mean: range, $100-$195). For all ART regimens, patients were projected to reach catastrophic coverage between February and May. For PrEP, monthly out-of-pocket costs were high in the coverage phase (mean [SD], $480 [$70]) and gap phase (mean, $430) before reaching the catastrophic phase (mean, $90) in May.

**Table 1.** Antiretroviral Therapy and Preexposure Prophylaxis Prices and Patient Out-of-Pocket Cost During Each Phase of a 2019 Medicare Part D Plan^a^

Drug regimen	Cost per 30 d, mean (SD), $	Patient out-of-pocket cost per 30 d, mean (SD), $			Coinsurance, %	Months in each Part D phase^b^		
		Coverage	Gap^c^	Catastrophic^c^		Coverage	Gap	Catastrophic
Antiretroviral therapy^d^								
BIC, TAF, and FTC	3090 (90)	910 (160)	770	155	29.7	Jan-Feb	Feb-Mar	Mar-Dec
DTG, ABC, and 3TC	2950 (100)	870 (150)	740	148	29.7	Jan-Feb	Feb-Mar	Mar-Dec
DTG+TAF and FTC	3490 (120)	980 (140)	870	175	28.1	Jan-Feb	Feb-Mar	Mar-Dec
RAL+TAF and FTC	3240 (100)	900 (140)	810	162	28.1	Jan-Feb	Feb-Mar	Mar-Dec
EVG, C, TDF, and FTC	3160 (100)	930 (160)	790	158	29.7	Jan-Feb	Feb-Mar	Mar-Dec
EVG, C, TAF, and FTC	3010 (100)	840 (120)	750	151	28.1	Jan-Feb	Feb-Mar	Mar-Dec
RAL+ABC and 3TC	2120 (350)	560 (160)	600	106	29.0	Jan-Feb	Feb-May	May-Dec
DRV, C, TAF, and FTC	3900 (110)	1160 (210)	970	195	29.8	Jan	Jan-Feb	Feb-Dec
DRV, R+TAF, and FTC	3690 (120)	1060 (170)	920	185	28.9	Jan-Feb	Feb-Mar	Mar-Dec
ATV, C+TAF, and FTC	3350 (110)	960 (160)	840	168	28.9	Jan-Feb	Feb-Mar	Mar-Decr
DRV, R+ABC, and 3TC	2560 (370)	720 (190)	710	128	29.8	Jan-Feb	Feb-Apr	Apr-Dec
DOR, TDF, and 3TC	2200 (60)	650 (120)	550	110	29.8	Jan-Feb	Mar-Apr	Apr-Dec
EFV, TDF, and FTC	2790 (90)	780 (110)	700	140	28.1	Jan-Feb	Feb-Mar	Mar-Dec
RPV, TAF, and FTC	2740 (90)	770 (110)	690	137	28.1	Jan-Feb	Feb-Mar	Mar-Dec
RPV, TDF, and FTC	2740 (90)	810 (140)	680	137	29.8	Jan-Feb	Feb-Mar	Mar-Dec
DTG+3TC	2000 (140)	550 (100)	530	100	30.0	Jan-Feb	Feb-May	May-Dec
DRV and R+RAL	3500 (110)	1010 (170)	880	175	28.9	Jan-Feb	Feb-Mar	Mar-Dec
DRV and R+3TC	2190 (150)	640 (130)	580	110	30.8	Jan-Feb	Feb-Apr	Apr-Dec
Median price	2980	860	750	149	29.4	Jan-Feb	Feb-Mar	Mar-Dec
Preexposure prophylaxis								
TDF and FTC	1710 (60)	480 (70)	430	90	28.1	Jan-Mar	Mar-May	May-Dec
TAF and FTC	1710 (60)	480 (70)	430	90	28.1	Jan-Mar	Mar-May	May-Dec

Abbreviations: 3TC, lamivudine; ABC, abacavir; ATV, atazanavir; BIC, bictegravir; C, cobicistat; DOR, doravirine; DRV, darunavir; DTG, dolutegravir; EFV, efavirenz; EVG, elvitegravir; FTC, emtricitabine; R, ritonavir; RAL, raltegravir; RPV, rilpivirine; TAF, tenofovir alafenamide; TDF, tenofovir disoproxil fumarate.

^a^ Standard 2019 Part D benefit: (1) $415 deductible; (2) coverage phase: patients pay a percentage of drug’s full list price (coinsurance), and the insurance plan pays the remainder; (3) coverage gap: starts at $3820 in total drug cost, brand-name drug cost is shared between the patient (25%), manufacturer (70%), and insurance plan (5%), and generic drug cost (lamivudine and abacavir-lamivudine) is shared between the patient (37%) and insurance plan (63%); and (4) catastrophic coverage: starts at $5100 in out-of-pocket costs (including manufacturer contribution during the gap), with costs shared by the patient (5%), insurance plan (15%), and Medicare (80%) for the rest of the year.

^b^ Patients may start the month in one phase, but end the month in a different phase.

^c^ Out-of-pocket costs in the gap phase equals 25% of drug price and in the catastrophic phase equals 5% of drug price. As such, SDs are not presented.

^d^ For regimens allowing either TAF and FTC or TDF and FTC, data are presented for TAF and FTC.

Under a standard 2019 Part D plan for 1 year of ART (price range, $24 010-$46 770; median, $35 780), patients without subsidies were projected to contribute 9% to 14% ($3270-$4350), insurance plans 18% to 24% ($5340-$8450), manufacturers 6% to 11% ($2370-$2750), and Medicare 53% to 67% ($12 770-$31 270) (Table 2). For 1 year of PrEP (price of both regimens ranges from $20 560 to $20 570), patients would contribute 15% ($2990), insurance plans 22% ($4570), manufacturers 13% ($2750), and Medicare 50% ($10 260). For a median-priced ART or PrEP regimen, monthly out-of-pocket costs would vary widely throughout the year as patients passed through each Part D phase (Figure). For patients with full LIS subsidies, Medicare would pay 67% to 76% of ART costs and 65% of PrEP costs.

**Table 2.** Distribution of Antiretroviral Therapy and Preexposure Prophylaxis Cost for 1 Year Under a Standard 2019 Medicare Part D Plan

Drug regimen	Annual Price, $	Annual price, $ (%)^a^			
		Patient	Insurance plan	Manufacturer	Medicare
Antiretroviral therapy					
BIC, TAF, and FTC	37 080	3860 (10)	7000 (19)	2710 (7)	23 510 (63)
DTG, ABC, and 3TC	35 430	3780 (11)	6760 (19)	2710 (8)	22 190 (63)
DTG+TAF and FTC	41 900	4060 (10)	7770 (19)	2750 (7)	27 320 (65)
RAL+TAF and FTC	38 890	3900 (10)	7320 (19)	2750 (7)	24 910 (64)
EVG, C, TDF, and FTC	37 900	3900 (10)	7120 (19)	2710 (7)	24 170 (64)
EVG, C, TAF, and FTC	36 130	3770 (10)	6910 (19)	2750 (8)	22 700 (63)
RAL+ABC and 3TC	25 420	3490 (14)	6040 (24)	2370 (9)	13 520 (53)
DRV, C, TAF, and FTC	46 770	4350 (9)	8450 (18)	2710 (6)	31 270 (67)
DRV, R+TAF, and FTC	44 230	4200 (9)	8090 (18)	2730 (6)	29 210 (66)
ATV, C+TAF, and FTC	40 250	4000 (10)	7500 (19)	2730 (7)	26 030 (65)
DRV, R+ABC, an d3TC	30 760	3760 (12)	6640 (22)	2460 (8)	17 900 (58)
DOR, TDF, an d3TC	26 390	3330 (13)	5400 (20)	2710 (10)	14 960 (57)
EFV, TDF, and FTC	33 420	3630 (11)	6500 (19)	2750 (8)	20 540 (61)
RPV, TAF, and FTC	32 880	3610 (11)	6420 (20)	2750 (8)	20 110 (61)
RPV, TDF, and FTC	32 860	3650 (11)	6370 (19)	2710 (8)	20 140 (61)
DTG+3TC	24 010	3270 (14)	5340 (22)	2630 (11)	12 770 (53)
DRV and R+RAL	42 000	4080 (10)	7760 (18)	2730 (7)	27 420 (65)
DRV and R+3TC	26 330	3410 (13)	5620 (21)	2610 (10)	14 690 (56)
Median price	35 780	3770 (11)	6830 (19)	2710 (8)	22 450 (63)
Preexposure prophylaxis					
TDF and FTC	20 570	2990 (15)	4570 (22)	2750 (13)	10 260 (50)
TAF and FTC	20 560	2990 (15)	4570 (22)	2750 (13)	10 250 (50)

Abbreviations: 3TC, lamivudine; ABC, abacavir; ATV, atazanavir; BIC, bictegravir; C, cobicistat; DOR, doravirine; DRV, darunavir; DTG, dolutegravir; EFV, efavirenz; EVG, elvitegravir; FTC, emtricitabine; R, ritonavir; RAL, raltegravir; RPV, rilpivirine; TAF, tenofovir alafenamide; TDF, tenofovir disoproxil fumarate.

^a^ Cost paid by patient, insurance plan, manufacturer, and Medicare may not sum up exactly to price owing to rounding to nearest $10. Percentages do not sum to 100% owing to rounding.

**Figure.**
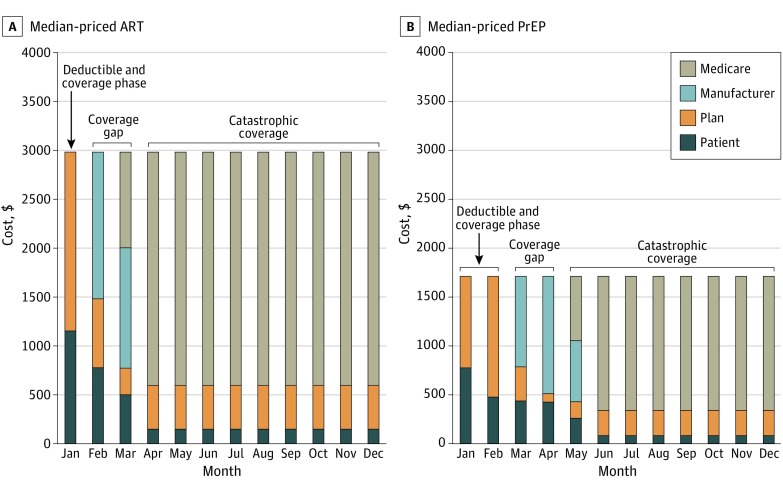
Part D Cost-Sharing Between Patients, Insurance Plans, Manufacturers, and Medicare for Median-Priced Antiretroviral Therapy (ART) and Preexposure Prophylaxis (PrEP)

## Discussion

In this national analysis, despite Medicare’s mandate that Part D insurance plans cover ART and PrEP, high drug prices mean that patients face out-of-pockets costs of $3000 to $4000 annually under a standard benefit. Such cost-sharing indicates that for beneficiaries, affordable access to ART or PrEP may depend on them receiving taxpayer subsidies to lower out-of-pocket costs. In addition to highlighting the cost burden to patients, we found that most of the remaining ART and PrEP costs in Part D are borne by Medicare and taxpayers, rather than insurance plans or manufacturers.

For Medicare beneficiaries with HIV, high out-of-pocket cost may be associated with decreased treatment adherence and with both individual- and population-level harm. Other studies have shown that out-of-pocket cost can have a negative association with the use of ART and PrEP.^6,10,11^ To the extent that affordability is associated with adherence, these results have important clinical and policy implications; the federal Ending the HIV Epidemic initiative will be successful only if patients have reliable access to HIV treatment and prevention. Patients’ high out-of-pocket costs are associated with the large expense of ART and PrEP and how Part D designs cost-sharing for patients. In 2019, nearly all Part D insurance plans structured patient cost-sharing for ART and PrEP as a percentage of drug price rather than a fixed-dollar copayment. Such coinsurance is also applied during the coverage gap and catastrophic phases. Thus, patients pay a percentage of ARV prices during all Part D phases, and higher ARV prices will lead to greater out-of-pocket costs.^16^ In addition, Part D varies cost-sharing in each coverage phase—deductible (100% coinsurance), coverage phase (median coinsurance of 29% for ART and 28% for PrEP), coverage gap phase (25%), and catastrophic phase (5%).^16^ This variation causes sizable month-to-month fluctuation in patients’ out-of-pocket costs. Beneficiaries have high copayments (≥$500) during the first few months of each year and still face monthly copayments of $100 to $195 even during catastrophic coverage. Consideration should be given to redesigning Part D to focus on patients’ out-of-pocket costs in predictable and stable dollar terms rather than as a changing percentage of drug price; this new design should also include a ceiling for patient out-of-pocket expenses.

Out-of-pocket costs for ARV will become even more important as more HIV beneficiaries age into Medicare, rather than qualifying based on disability, which often brings support through LIS. In 2014, of the Medicare beneficiaries 65 years or older with HIV, two-thirds initially qualified for Medicare based on age.^15^ Nearly half of people with HIV are 50 years or older, and Medicare will be a critical source of coverage for this population in the decade ahead.^15,23^^,^ With the aging HIV population comes the need for PrEP access for older patients. In 2018, approximately 17% of new HIV infections in the United States occurred in persons 50 years or older,^3^ and in 2016, 7% of individuals using PrEP were 55 years or older.^24^ Over time, these individuals with indications for PrEP will increasingly enter Medicare owing to their age, without necessarily qualifying for LIS.

For beneficiaries with HIV who qualify for LIS, out-of-pocket costs are lower. Beneficiaries with dual eligibility with Medicaid or who have low income and assets qualify for full LIS, and cost-sharing is limited to a few dollars for drugs.^22^ However, beneficiaries with only partial LIS due to higher income (between 135% and 150% of the federal poverty level) are required to pay 15% of drug costs. These individuals may still receive low-income subsidies from other federally or state-funded programs, such as the Ryan White AIDS Drug Assistance Program, which further subsidizes out-of-pocket costs for HIV care not covered by other insurance.^25^ In either case, the cost burden of ART and PrEP shifts further to the taxpayer. For patients with full LIS, Medicare contributes up to three-fourths of annual ARV cost under a 2019 standard Part D benefit. Even for patients without LIS, Medicare pays half to two-thirds of ARV cost because high prices push patients quickly into catastrophic coverage, in which Medicare assumes 80% of the cost burden. Federal law prohibits Medicare from negotiating directly with manufacturers for lower drug prices.^21^ Furthermore, both patient and Medicare contributions are calculated based on a drug’s full list price without reflecting the rebates that insurance plans receive from manufacturers or pharmacy benefit managers.^21,26,27,28^ Thus, as drug prices continue to increase, the cost assumed by patients and Medicare (taxpayers) will increase correspondingly. In 2017, Part D expenditures for ARVs exceeded $4 billion,^14^ which could increase substantially as efforts to expand the use of ART and PrEP proceed.

As currently designed, Part D insurance plans would cover less than one-fourth of the cost of ART and PrEP. These calculations exclude rebates and discounts from manufacturers and pharmacy benefit managers. Insurance plans’ actual contributions are likely lower, although their exact magnitude remains unknown because rebate information is proprietary. For manufacturers, their 70% price discount during the coverage gap represented only a modest contribution (≤$2750) and was frequently 10% or less of ARV prices.

Proposed legislation would redesign Part D to lower patients’ cost-sharing by capping annual out-of-pocket costs or passing discounts and rebates directly to patients.^16,21,26,27,28^ Others advocate shifting a greater proportion of cost away from Medicare during the catastrophic coverage phase and onto insurance plans (50%) and manufacturers (30%).^26,27^ However, there is concern that doing so may increase premiums and may increase drug prices further. Thus, we believe that there is a need to address ART and PrEP price directly, such as allowing Medicare to negotiate drug prices, imposing penalties if drug prices increase beyond the general inflation rate,^13,16^ setting US prices based on lower prices in other countries, and recouping government-funded research support in the case of PrEP development.^29^ Our findings suggest that current ART and PrEP costs fall largely on patients and taxpayers, which is not conducive to the success of the Ending the HIV Epidemic initiative. It seems to be apparent that achieving a 90% incidence reduction in HIV by 2030 will require legislation to manage the price of these drugs.

### Limitations

This study has some limitations. We projected annual cost-sharing based only on use of each ART or PrEP and no other drugs under a standard 2019 Part D insurance plan. Actual out-of-pocket cost would depend on insurance plan–specific benefit parameters (eg, deductibles and terms for cost-sharing) as well as the cost of beneficiaries’ other prescriptions.

## Conclusions

The high prices of ART and PrEP and the design of Medicare Part D mean that patients can face thousands of dollars in out-of-pocket cost for drugs for HIV treatment and prevention. Thus, many beneficiaries with Part D may struggle to afford access to ART and PrEP unless they receive taxpayer-funded subsidies. Furthermore, Medicare (taxpayers), rather than health insurance plans or manufacturers, appears to shoulder much of the remaining cost burden. We believe that to realize Ending the HIV Epidemic initiative goals, reforms must quickly and effectively address the high price of ARVs as well as redesign Part D cost-sharing.

## References

[1] HIV.gov US statistics. Accessed February 6, 2020. https://www.hiv.gov/hiv-basics/overview/data-and-trends/statistics

[2] Harris NS, Johnson AS, Huang YA, Vital signs: status of human immunodeficiency virus testing, viral suppression, and HIV preexposure prophylaxis—United States, 2013-2018. MMWR Morb Mortal Wkly Rep. 2019;68(48):-. doi:10.15585/mmwr.mm6848e1 31805031 PMC6897528

[3] Centers for Disease Control and Prevention. HIV surveillance report. Accessed February 6, 2020. https://www.cdc.gov/hiv/statistics/overview/index.html

[4] HIV.gov What is “Ending the HIV Epidemic: A Plan for America”? Accessed February 6, 2020. https://www.hiv.gov/federal-response/ending-the-hiv-epidemic/overview

[5] Centers for Disease Control and Prevention. PrEP. Accessed February 6, 2020. https://www.cdc.gov/hiv/basics/prep.html

[6] US Department of Health and Human Services Guidelines for the use of antiretroviral agents in adults and adolescents with HIV: cost considerations and antiretroviral therapy. Updated December 18, 2019. Accessed February 6, 2020. https://aidsinfo.nih.gov/guidelines/html/1/adult-and-adolescent-arv/459/cost-considerations-and-antiretroviral-therapy

[7] McCann NC, Horn TH, Hyle EP, Walensky RP HIV antiretroviral therapy costs in the United States, 2012-2018. JAMA Intern Med. 2020. Published online February 3, 2020. doi:10.1001/jamainternmed.2019.7108 32011622 PMC7042880

[8] Beer L, Tie Y, Weiser J, Shouse RL Nonadherence to any prescribed medication due to cost among adults with HIV infection—United States, 2016-2017. MMWR Morb Mortal Wkly Rep. 2019;68(49):1129-1133. doi:10.15585/mmwr.mm6849a1 31830009 PMC6919290

[9] Belenky N, Pence BW, Cole SR, Associations between Medicare Part D and out-of-pocket spending, HIV viral load, adherence, and ADAP use in dual eligibles with HIV. Med Care. 2018;56(1):47-53. doi:10.1097/MLR.0000000000000843 29227443 PMC5728680

[10] Whitfield THF, John SA, Rendina HJ, Grov C, Parsons JT Why I quit pre-exposure prophylaxis (PrEP)? a mixed-method study exploring reasons for PrEP discontinuation and potential re-initiation among gay and bisexual men. AIDS Behav. 2018;22(11):3566-3575. doi:10.1007/s10461-018-2045-1 29404756 PMC6077114

[11] Golub SA, Myers JE Next-wave HIV pre-exposure prophylaxis implementation for gay and bisexual men. AIDS Patient Care STDS. 2019;33(6):253-261. doi:10.1089/apc.2018.0290 31094576 PMC6588121

[12] Hwang TJ, Dusetzina SB, Feng J, Maini L, Kesselheim AS Price increases of protected-class drugs in Medicare Part D, relative to inflation, 2012-2017. JAMA. 2019;322(3):267-269. doi:10.1001/jama.2019.7521 31310287 PMC6635901

[13] Kates J, Dawson L, Cubanski J Quick look: antiretroviral price increases in Medicare Part D. Kaiser Family Foundation. Published December 17, 2019. Accessed February 6, 2020. https://www.kff.org/hivaids/issue-brief/quick-look-antiretroviral-price-increases-in-medicare-part-d/#

[14] Centers for Medicare & Medicaid Services Medicare Part D drug spending dashboard & data. Accessed February 6, 2020. https://www.cms.gov/Research-Statistics-Data-and-Systems/Statistics-Trends-and-Reports/Information-on-Prescription-Drugs/MedicarePartD

[15] Kaiser Family Foundation Medicare and HIV. Published October 14, 2016. Accessed February 6, 2020. https://www.kff.org/hivaids/fact-sheet/medicare-and-hiv/

[16] Kaiser Family Foundation An overview of the Medicare Part D prescription drug benefit. Published November 13, 2019. Accessed February 6, 2020. https://www.kff.org/medicare/fact-sheet/an-overview-of-the-medicare-part-d-prescription-drug-benefit/

[17] Yazdany J, Dudley RA, Lin GA, Chen R, Tseng CW Out-of-pocket costs for infliximab and its biosimilar for rheumatoid arthritis under Medicare Part D. JAMA. 2018;320(9):931-933. doi:10.1001/jama.2018.7316 30193264 PMC6142992

[18] Dusetzina SB, Huskamp HA, Keating NL Specialty drug pricing and out-of-pocket spending on orally administered anticancer drugs in Medicare Part D, 2010 to 2019. JAMA. 2019;321(20):2025-2027. doi:10.1001/jama.2019.4492 31135837 PMC6547115

[19] Centers for Medicare & Medicaid Services Prescription drug plan formulary, pharmacy network, and pricing information files. Accessed February 6, 2020. https://www.cms.gov/Research-Statistics-Data-and-Systems/Files-for-Order/NonIdentifiableDataFiles/PrescriptionDrugPlanFormularyPharmacyNetworkandPricingInformationFiles

[20] US Department of Health and Human Services Guidelines for the use of antiretroviral agents in adults and adolescents with HIV: what to start: initial combination regimens for the antiretroviral-naive patient. Updated December 18, 2019. Accessed February 6, 2020. https://aidsinfo.nih.gov/guidelines/html/1/adult-and-adolescent-arv/11/what-to-start

[21] Frank RG, Nichols LM Medicare drug-price negotiation—why now…and how. N Engl J Med. 2019;381(15):1404-1406. doi:10.1056/NEJMp1909798 31483959

[22] Center for Benefits Access Full low-income subsidy (LIS)/extra help (2020)—48 states + DC. Accessed February 6, 2020. https://www.ncoa.org/wp-content/uploads/part-d-lis-eligibility-and-benefits-chart.pdf

[23] Centers for Disease Control and Prevention HIV among people aged 50 and older. Accessed February 6, 2020. https://www.cdc.gov/hiv/group/age/olderamericans/index.html

[24] Huang YA, Zhu W, Smith DK, Harris N, Hoover KW HIV preexposure prophylaxis, by race and ethnicity—United States, 2014-2016. MMWR Morb Mortal Wkly Rep. 2018;67(41):1147-1150. doi:10.15585/mmwr.mm6741a3 30335734 PMC6193685

[25] Kaiser Family Foundation. AIDS drug assistance programs (ADAPs). Published August 16, 2017. Accessed February 6, 2020. https://www.kff.org/hivaids/fact-sheet/aids-drug-assistance-programs/

[26] Dusetzina SB, Keating NL, Huskamp HA Proposals to redesign Medicare Part D—easing the burden of rising drug prices. N Engl J Med. 2019;381(15):1401-1404. doi:10.1056/NEJMp1908688 31483987

[27] Cubanski J, Neuman T How will the Medicare Part D benefit change under current law and leading proposals? Kaiser Family Foundation. Published October 11, 2019. Accessed February 6, 2020. https://www.kff.org/medicare/issue-brief/how-will-the-medicare-part-d-benefit-change-under-current-law-and-leading-proposals/

[28] Gellad WF, Ennis M, Kuza CC A new safe harbor—turning drug rebates into discounts in Medicare Part D. N Engl J Med. 2019;380(18):1688-1690. doi:10.1056/NEJMp1902692 30946552

[29] US Department of Health and Human Services. United States files patent infringement lawsuit against Gilead related to Truvada and Desovy for pre-exposure prophylaxis of HIV. Published November 6, 2019. Accessed February 6, 2020. https://www.hhs.gov/about/news/2019/11/06/us-files-patent-infringement-lawsuit-against-gilead-pre-exposure-prophylaxis-hiv.html

